# Prediction of cyanidin 3-rutinoside content in *Michelia crassipes* based on near-infrared spectroscopic techniques

**DOI:** 10.3389/fpls.2024.1346192

**Published:** 2024-05-03

**Authors:** Yuguang Xiao, Xiaoshu Zhang, Jun Liu, He Li, Jingmin Jiang, Yanjie Li, Shu Diao

**Affiliations:** ^1^ Research Institute of Subtropical Forestry, Chinese Academy of Forestry, Hangzhou, China; ^2^ School of Civil Engineering and Architecture, Xinxiang University, Xinxiang, China; ^3^ Research Institute of Landscape Plants, Guizhou Academy of Forestry, Guiyang, China

**Keywords:** model calibration, NIR spectroscopy, regression algorithm, cyanidin 3-rutinoside, *Michelia crassipes*

## Abstract

Currently the determination of cyanidin 3-rutinoside content in plant petals usually requires chemical assays or high performance liquid chromatography (HPLC), which are time-consuming and laborious. In this study, we aimed to develop a low-cost, high-throughput method to predict cyanidin 3-rutinoside content, and developed a cyanidin 3-rutinoside prediction model using near-infrared (NIR) spectroscopy combined with partial least squares regression (PLSR). We collected spectral data from *Michelia crassipes* (Magnoliaceae) tepals and used five different preprocessing methods and four variable selection algorithms to calibrate the PLSR model to determine the best prediction model. The results showed that (1) the PLSR model built by combining the blockScale (BS) preprocessing method and the Significance multivariate correlation (sMC) algorithm performed the best; (2) The model has a reliable prediction ability, with a coefficient of determination (R^2^) of 0.72, a root mean square error (RMSE) of 1.04%, and a residual prediction deviation (RPD) of 2.06. The model can be effectively used to predict the cyanidin 3-rutinoside content of the perianth slices of *M. crassipes*, providing an efficient method for the rapid determination of cyanidin 3-rutinoside content.

## 1 Introduction

*Michelia crassipes* Y.W. Law is an evergreen shrub or small tree, the only purple-flowered species in the genus *Michelia*, sporadically distributed in Guangdong, Hunan, Guangxi, Jiangxi, Guizhou and other provinces of China, and grows in dense forests on mountain slopes and in ravines at an altitude of 300-1000 m (Committee FoCE, 1996; Liu et al., 2002; Yang et al., 2003). The flower color of genus *Michelia* is mostly white or yellowish, while the tepals of *M. crassipes* are purplish-red or deep purple, so it is often used as an important parent for the improvement of the flower color of genus *Michelia* and is an excellent resource for flower viewing and flower color breeding (Liao, 2007; Shao et al., 2015b; Shao et al., 2015a; Shao et al., 2016; Chai et al., 2018).

Anthocyanins are a class of flavonoid that are widely found in plants in nature. These anthocyanins are multi-functional and can play an important role in protecting against ultraviolet radiation, coping with drought and fighting pathogens (Tohge and Fernie, 2017). As a water-soluble natural pigment, anthocyanins appear blue in alkaline cellular fluids and red under acidic conditions. Therefore, many plant organs such as flowers, leaves, and fruits appear purple, red, or blue, with a positive correlation between the shade of color and anthocyanin content (Tanaka et al., 2008; Li et al., 2014). Cyanidin 3-rutinoside (Cy3R), the main component of anthocyanins in the tepals of *M. crassipes*, plays an important role in the formation of purple color in the tepals of *M. crassipes* (Liu et al., 2020b). Previous studies have found that *M. crassipes* exhibits significant genetic diversity, with tepals of different individuals differing in color, all showing a purple hue (He et al., 2018; Xiao et al., 2023). The correlation between flower color phenotype and Cy3R content is expected to provide important basic information for revealing the mechanism of flower color formation in plants and related genetic analysis.

There are many traditional methods used to detect anthocyanins content in plant tissue, such as microwave method, pH differential method and high performance liquid chromatography (Lee et al., 2005; Chen et al., 2007; Rong et al., 2016). The results of these traditional methods are accurate, but they are time-consuming and cumbersome as they require a lot of labor and material resources during the experimental process (Dzhanfezova et al., 2020). In recent years, High-performance liquid chromatography (HPLC) has begun to be gradually used for the determination of anthocyanins content (Kim and Lee, 2020; Thuy et al., 2021), which is fast and simple to operate, but requires expensive instrumentation and cannot be quickly detected in the field (Liu et al., 2022). In addition, all of these methods require sample destruction, which makes it difficult to achieve non-destructive detection and has a certain impact on the environment (Firmani et al., 2019). Therefore, it is of great significance to develop simpler, rapid, and non-destructive methods for the determination of anthocyanins content.

Near-Infrared (NIR) spectroscopy is a fast, easy-to-use and non-destructive detection technique (Wetzel, 1998; Zhang et al., 2023) which utilizes the spectral information in the near-infrared wavelength band (800 - 2500 nm) to obtain chemical and structural information about a specimen (Rinnan and Rinnan, 2007). The origin of this technique dates back to the late 1850s (Butler, 1983). With continuous development and maturation, NIR spectroscopy is now widely used in the fields of food, medicine, agriculture and industry (Biancolillo et al., 2019; Abu-Khalaf and Hmidat, 2020; Prananto et al., 2020; Rossi and Lozano, 2020; Li et al., 2023; Trenfield et al., 2023). In recent years, researchers have begun to apply NIR spectroscopy to forestry. For example, Y Zhang, Q Luan, J Jiang and Y Li (Zhang et al., 2021) utilized near-infrared (NIR) spectroscopy combined with partial least squares regression (PLSR) to predict the malondialdehyde (MDA) content of slash pine needles in a real-time and rapid manner to understand plant stress. In addition, Zhang et al. (2023) utilized near-infrared (NIR) spectroscopy to non-destructively detect the sugar content of peach under various conditions.

NIR spectroscopic data can be obtained from NIR instruments. These data contain a lot of information about the physical and chemical properties of the molecules (Czarnecki et al., 2021). These data provide a valuable resource for analysis, but they are also accompanied by noise interference (Liu et al., 2020a). To effectively eliminate noise, preprocessing spectral data becomes a critical step in constructing chemometric models (Katsumoto et al., 2001). In addition, choosing appropriate variables (bands) can significantly improve the model performance (Ma et al., 2018). However, no studies have been reported on the prediction of anthocyanin content of *M. crassipes* tepals.

Therefore, the aim of this study was to (1) establish a model for predicting the content of cyanidin 3-rutinoside in *M. crassipes* tepals with the help of near-infrared spectroscopy combined with chemometrics; and (2) compare the model performance of different combinations of spectral preprocessing and variable selection methods. The established model for predicting the content of cyanidin 3-rutinoside can not only realize the rapid acquisition of the flower color phenotype of *M. crassipes*, but also provide a reference for the rapid and non-destructive detection of the content of cyanidin 3-rutinoside in other plant species.

## 2 Materials and methods

### 2.1 Plant materials

The plant materials used in this experiment were obtained from the germplasm resource nursery of the Chinese Academy of Forestry Research Institute of Subtropical Forestry (30° 3’ N, 119° 57’ E) and Guizhou Academy of Forestry (26° 30’ N, 106° 44’ E). Based on the results of the previous flower color survey of *M. crassipes* resources in the two locations, *M. crassipes* individuals with large differences in flower color were randomly selected. Samples were collected in the morning of April-May 2023 when the weather was clear. *M. crassipes* flowers at the bud stage (flower buds enlarged, bracts dehiscent, showing purple tepals) and at blooming stage (both rounds of tepals unfolded, with a large amount of pollen dispersed, but not browning and withering) were plucked together with their pedicels, and then wrapped around the pedicels at the point of fracture with wet paper towels, and carefully put into air-filled self-sealing bags, to prevent the petals from falling off by squeezing (Fu and Dai, 2016; Yuan et al., 2023). A total of 66 samples were brought back to the laboratory for NIR spectroscopy. The collected samples were stored in a refrigerator at -80°C for the subsequent determination of cyanidin 3-rutinoside content.

### 2.2 Determination of monomeric anthocyanin content

Spectrophotometric method, is considered as a valid alternative to HPLC method due to its simplicity, rapidity and economy (Lee et al., 2008). This method is similar to HPLC method in terms of accuracy of results (Lao and Giusti, 2016), therefore, spectrophotometric method was used in this study for the determination of anthocyanin content. Pre-prepared 1% hydrochloric acid-methanol solution for anthocyanin extraction was obtained as follows: 3 ml of 36% concentrated hydrochloric acid was aspirated with a pipette gun and fixed to 100 ml with methanol (Lin et al., 2011). Accurately weighed 0.25 g of the sample was cut into 10 ml centrifuge tubes, replenished with 1% hydrochloric acid-methanol solution to 8 ml, and extracted at a low temperature and protected from light at 4 °C for 48 h, during which time the centrifuge tubes were shaken 2-3 times. A 96-well plate was prepared with 1% hydrochloric acid-methanol solution as a blank control, and 200 μl of anthocyanin extract was taken, and the absorbance value was read at 530 nm with a microplate reader (SpectraMax iD5, Molecular Devices, USA), and three replicates were set for each sample. The standard curve was plotted by gradient dilution with cyanidin 3-rutinoside standard (≥95%) (Shanghai Yuanye Biotechnology Co., Ltd.). The content of cyanidin 3-rutinoside was calculated using the following formula:


$$\text{Cyanidin }3-\text{rutinoside of tissue sample }(\text{mg }{\text{g}}^{-1})=(\text{C}\times {\text{V}}_{\text{T}})/(\text{W}\times {\text{V}}_{1})$$

Where: C = content of cyanidin 3-rutinoside (mg ml^-1^) in the measuring tube obtained from the standard curve; V_T_ = total volume of anthocyanin extract (ml) = 8; V_1_ = volume of anthocyanin crude extract used in the addition of the sample (ml); W = fresh weight of the sample (g).

### 2.3 NIR spectrum measurements

Spectral raw data were determined using a portable near-infrared spectral analyzer (LF-2500, Spectral evolution, USA). The spectral range was 1000-2500 nm with a resolution of 6 nm. The outer petals of the collected petals were placed on the background board, and the handheld fiber-optic contact probe was used to directly scan the petals at different flower colors. In order to minimize noise contamination and to ensure accuracy, the probe was closely attached to the petal surface during the measurement, while standard whiteboard correction was performed in time. A total of 129 spectral data were measured. From the 129 spectral data, 103 data were randomly selected as the calibration set and 26 data as the validation set.

### 2.4 Spectral analysis methods

Spectra typically have a relatively low signal-to-noise ratio in this region of 2400-2500 nm, and this spectral region was removed in order to eliminate the effect of noise (Xu et al., 2018; Guo et al., 2021). Preprocessing of spectral data is necessary to further minimize the effects of instruments, probe offsets, and surroundings on spectral data and to maximize the spectral differences (Osborne et al., 1993; Qiu et al., 2022). In this study, six preprocessing methods were applied, namely Standard normal variate (SNV), Block scale (BS), Detrended variable (DET), and Block normalization (BN), Removal of polynomial trends and standard normal transformation (DET-SNV), Block scale and standard normal transformation (BS-SNV). Four variable selection methods are also applied: bounded variable elimination (bve) (Eén and Biere, 2005; Soos et al., 2020), genetic algorithm (ga) (Molajou et al., 2021), regularized elimination procedure, and rep) (Mehmood et al., 2011), Significance multivariate correlation (sMC) (Tran et al., 2014).

As a classical linear multivariate analysis algorithm, PLSR has been widely used in the field of spectral data modeling (Cheng and Sun, 2017). When the number of independent variables is large and multicollinearity exists among these independent variables, the use of traditional multiple regression methods may lead to a decrease in the predictive performance of the model (Ma et al., 2023; Yang et al., 2023). Also, in the face of a limited number of samples, traditional methods may increase the risk of overfitting. However, PLSR methods can address these challenges more effectively and provide a better way to solve the above problems. Therefore, in this study, we completed the construction of a prediction model for the content of cyanidin 3-rutinoside based on PLSR in combination with the above preprocessing methods. The number of latent variables (LVs) was optimized by Leave-one-out cross-validation (LOOCV). Meanwhile, we used the coefficient of determination (R^2^), the root mean square error (RMSE), residual prediction deviation (RPD) and number of LVs as metrics to evaluate the model performance (Jin et al., 2020; Hssaini et al., 2022). Among these metrics, the closer the R^2^ value is to 1, the better and more stable the model fit is. Whereas, the closer the RMSE value is to 0, the higher the RPD value is, the superior predictive performance of the model is indicated, and the number of LVs is less than 10 as much as possible to avoid overfitting the model (Guo et al., 2021; Hssaini et al., 2022). Identification of the spectral regions that have a significant impact on the model was performed by building the PLSR model in eight independent sessions. In each modeling, the dataset was randomly assigned and divided into a calibration set and a validation set in an 8:2 ratio.

### 2.5 Software tools

All data were completed analyzed on R software (v4.3.1). The R packages “pls” and “enpls” were used to construct the PLSR model; The “prospectr” package was used to manipulate NIR spectral data (Wehrens and Mevik, 2007; Stevens and Ramirez-Lopez, 2014; Xiao et al., 2019). All plotting was performed using the “ggplot2” package (Wickham, 2011).

## 3 Results

### 3.1 Features of spectra

**Figure 1:**
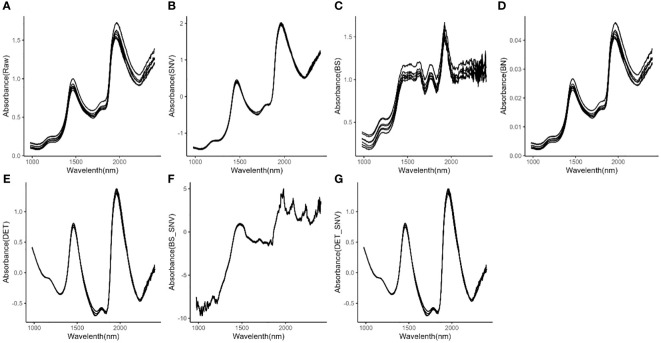
Spectra of *M. crassipes* tepals; **(A)** raw spectra; **(B)** SNV; **(C)** BS; **(D)** BN; **(E)** DET; **(F)** BS-SNV; **(G)** DET-SNV.

Selected raw spectra of eight representative *M. crassipes* tepals are shown in **Figure 1A**. The spectra after SNV, BS, BN, DET, BS-SNV and DET-SNV pretreatment are shown in **Figures 1B–G**, respectively. By observing the raw spectra, it was found that the samples exhibited significant absorption characteristic peaks near the bands of about 1400 nm and 2100 nm, and this observation was similar to the spectra after applying SNV, BN, DET, and DET-SNV preprocessing. However, the spectra after applying the BS and BS-SNV treatments show a greater number of peaks with sharper morphology, exhibiting more pronounced volatility. Additional absorption peaks were observed even in the originally relatively smooth spectral region.

### 3.2 Statistical values for cyanidin 3-rutinoside

**Figure 2:**
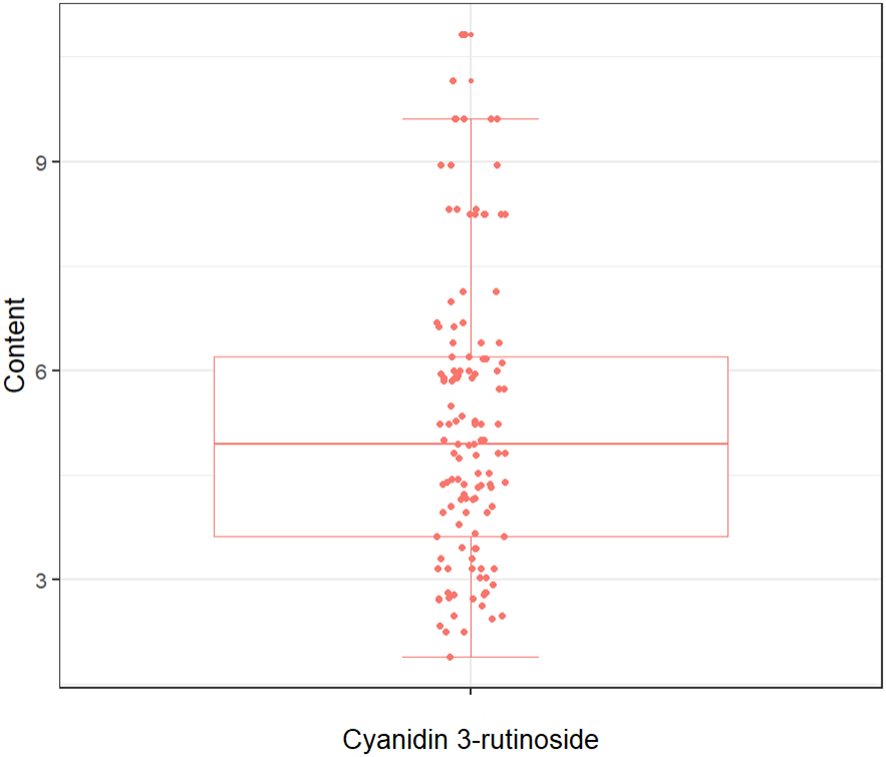
The boxplot of cyanidin 3-rutinoside content for *M. crassipes* samples. The boxes represent the interquartile range, the lines inside the boxes represent the medians, and the whiskers denote the lowest and highest values within 1.5 times the interquartile range. Each point indicates a value of cyanidin 3-rutinoside content.

The quantitative analysis conducted in this study on the concentration of cyanidin 3-rutinoside within the tepals of *M. crassipes* is graphically represented in **Figure 2**, where the minimum value was 1.89, the maximum value was 10.83, and the mean value was 5.25 with a standard deviation of 2.11. The determined values of Cy3R content showed a wide range of variation, a result that facilitates the calibration of the model.

### 3.3 Model performance

The effects of six different spectral data preprocessing methods with four variable selection strategies in PLSR models are summarized in **Table 1**, including performance metrics for both the calibration and validation sets. Among all models, the calibration set has an average R^2^ value of 0.68 and an average RMSE value of 1.18%, with the highest values of 0.68 (R^2^) and 1.20% (RMSE), and the lowest values of 0.67 (R^2^) and 1.16% (RMSE); while the validation set has an average R^2^ value of 0.73 and an average RMSE value of 1.03%, with the highest values of 0.75 (R^2^) and 1.09% (RMSE), and the lowest values were 0.69 (R^2^) and 1.01% (RMSE). In addition, the mean value of RPD values for all models was 1.65 with the highest value of 2.06 and the lowest value of 1.34; the number of LVs ranged between 3 and 15, with 13 models having a number of LVs greater than 10, which may be an overfitting phenomenon.

**Table 1:** Comparison of R^2^, RMSE and RPD values of calibration and validation sets of PLSR prediction models based on different spectral preprocessing and variable selection methods.

Pro-processing	Variable selection	Calibration				Validation				RPD	LV
		R^2^		RMSE(%)		R^2^		RMSE(%)			
		Mean	SD	Mean	SD	Mean	SD	Mean	SD		
OG	raw	0.67	0.04	1.19	0.09	0.75	0.07	1.01	0.19	1.65	9
	ga_sel	0.68	0.04	1.18	0.10	0.75	0.07	1.03	0.18	1.64	13
	rep_sel	0.67	0.04	1.18	0.10	0.74	0.06	1.05	0.18	1.69	9
	bve_sel	0.67	0.04	1.18	0.10	0.74	0.06	1.04	0.18	1.43	7
	sMC_sel	0.68	0.04	1.18	0.10	0.74	0.06	1.02	0.18	1.90	3
SNV	raw	0.68	0.04	1.18	0.10	0.74	0.06	1.04	0.18	1.48	9
	ga_sel	0.68	0.04	1.19	0.09	0.71	0.10	1.04	0.18	1.70	12
	rep_sel	0.67	0.04	1.19	0.10	0.75	0.06	1.03	0.18	1.40	10
	bve_sel	0.68	0.04	1.18	0.10	0.74	0.06	1.05	0.18	1.58	4
	sMC_sel	0.68	0.04	1.18	0.10	0.71	0.10	1.05	0.19	1.69	14
BS	raw	0.67	0.04	1.19	0.10	0.75	0.07	1.02	0.18	1.76	9
	ga_sel	0.68	0.04	1.18	0.10	0.72	0.08	1.05	0.18	1.83	15
	rep_sel	0.68	0.04	1.18	0.10	0.71	0.12	1.03	0.18	2.06	13
	bve_sel	0.68	0.04	1.17	0.11	0.73	0.07	1.07	0.20	1.88	7
	sMC_sel	0.68	0.04	1.18	0.10	0.72	0.09	1.04	0.18	2.06	9
BN	raw	0.67	0.04	1.18	0.10	0.75	0.07	1.02	0.18	1.60	9
	ga_sel	0.68	0.04	1.17	0.11	0.74	0.06	1.04	0.18	1.47	14
	rep_sel	0.67	0.04	1.19	0.10	0.75	0.06	1.03	0.18	1.73	13
	bve_sel	0.67	0.04	1.19	0.10	0.75	0.07	1.02	0.18	1.67	7
	sMC_sel	0.67	0.04	1.19	0.10	0.73	0.07	1.04	0.18	1.43	7
DET	raw	0.68	0.04	1.19	0.09	0.71	0.11	1.03	0.18	1.55	9
	ga_sel	0.68	0.04	1.19	0.10	0.73	0.07	1.02	0.18	1.73	11
	rep_sel	0.68	0.04	1.19	0.09	0.69	0.16	1.03	0.18	1.60	12
	bve_sel	0.67	0.04	1.20	0.10	0.73	0.07	1.02	0.18	1.34	12
	sMC_sel	0.67	0.04	1.19	0.10	0.75	0.06	1.04	0.18	1.40	11
BS_SNV	raw	0.67	0.04	1.19	0.09	0.75	0.06	1.02	0.18	1.89	9
	ga_sel	0.68	0.04	1.18	0.10	0.73	0.07	1.03	0.18	1.69	6
	rep_sel	0.68	0.05	1.16	0.12	0.72	0.10	1.08	0.23	1.57	6
	bve_sel	0.67	0.04	1.19	0.10	0.75	0.06	1.04	0.18	1.37	7
	sMC_sel	0.67	0.04	1.19	0.09	0.73	0.08	1.03	0.18	1.77	6
DET_SNV	raw	0.68	0.04	1.18	0.10	0.73	0.08	1.04	0.18	1.68	9
	ga_sel	0.68	0.04	1.19	0.10	0.74	0.06	1.02	0.18	1.80	4
	rep_sel	0.68	0.04	1.17	0.11	0.72	0.10	1.09	0.24	1.45	11
	bve_sel	0.67	0.04	1.20	0.10	0.75	0.07	1.01	0.19	1.83	5
	sMC_sel	0.67	0.04	1.19	0.10	0.73	0.07	1.06	0.19	1.48	11

PLS, partial least squares; R^2^, coefficient of determination; RMSE, root mean square error; RPD, residual prediction deviation; LV, latent variable; OG, original spectrum; SNV, standard normal variate; BS, block scale; BN: block normalization; DET, detrended variable; BS-SNV, block scale and standard normal variate; DET-SNV, detrended variable and standard normal variate; ga, genetic algorithm; rep, regularized elimination procedure; bve, bounded variable elimination; sMC, Significance multivariate correlation.

The performance of the models with SNV, DET and DET-SNV preprocessing methods was improved compared to the models without data preprocessing. Without the variable selection method, the model built by the DET-SNV preprocessing method had the highest performance with a calibration set R^2^ and RMSE of 0.68 and 1.18%, respectively, and an RPD value of 1.68. This was followed by the SNV, DET, and BN preprocessing methods. The BS and BS-SNV preprocessing methods had the worst model performance, with a calibration set R^2^ and RMSE were 0.67 and 1.19%, respectively.

When combining the four variable selection methods with all the preprocessing methods, the model performance was essentially similar. However, when combining the sMC variable selection methods with the BS preprocessing methods, the PLSR model performed best, with R^2^ and RMSE of 0.68 and 1.18% for the calibration set, and 0.72 and 1.04% for the validation set, with an RPD value of 2.06, and a number of LVs of 9.

### 3.4 Establishment of a predictive model for cyanidin 3-rutinoside content

**Figure 3:**
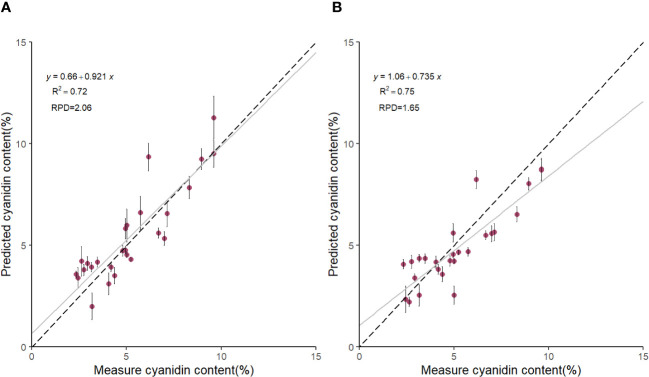
Scatterplot of predicted Cy3R content of *M. crassipes* tepals based on **(A)** block-scale-significance multivariate correlation (BS-sMC) algorithm combined with partial least squares regression (PLSR) modeling and **(B)** original full-length spectral PLSR modeling. The black dashed line indicates the predicted Cy3R values vs. measured values; the gray solid line is the linear regression line of the model; the error bars for each scatter indicate the prediction error obtained by eight random calibrations of the model.

**Figure 4:**
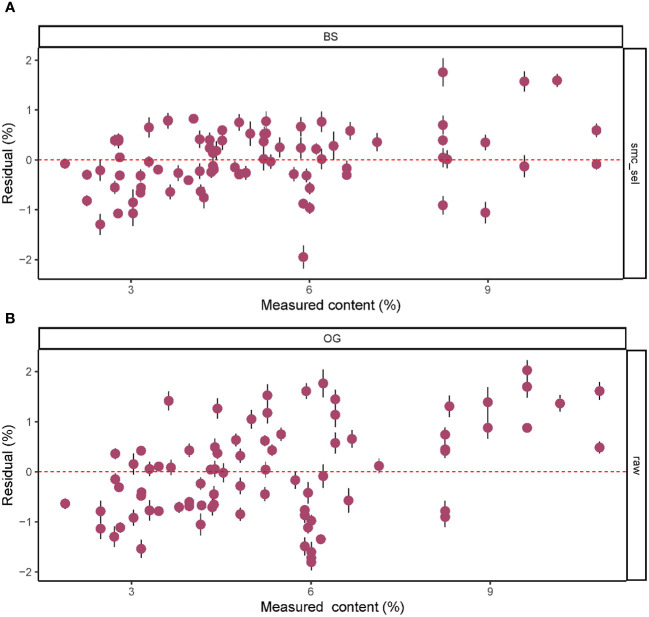
Residual plots of predicted *M. crassipes* Cy3R content based on **(A)** the BS-sMC algorithm PLSR model and **(B)** the original full-length spectral PLSR model. The error bars of the predicted values represent the SDs derived from the eight simulation models.

**Figure 5:**
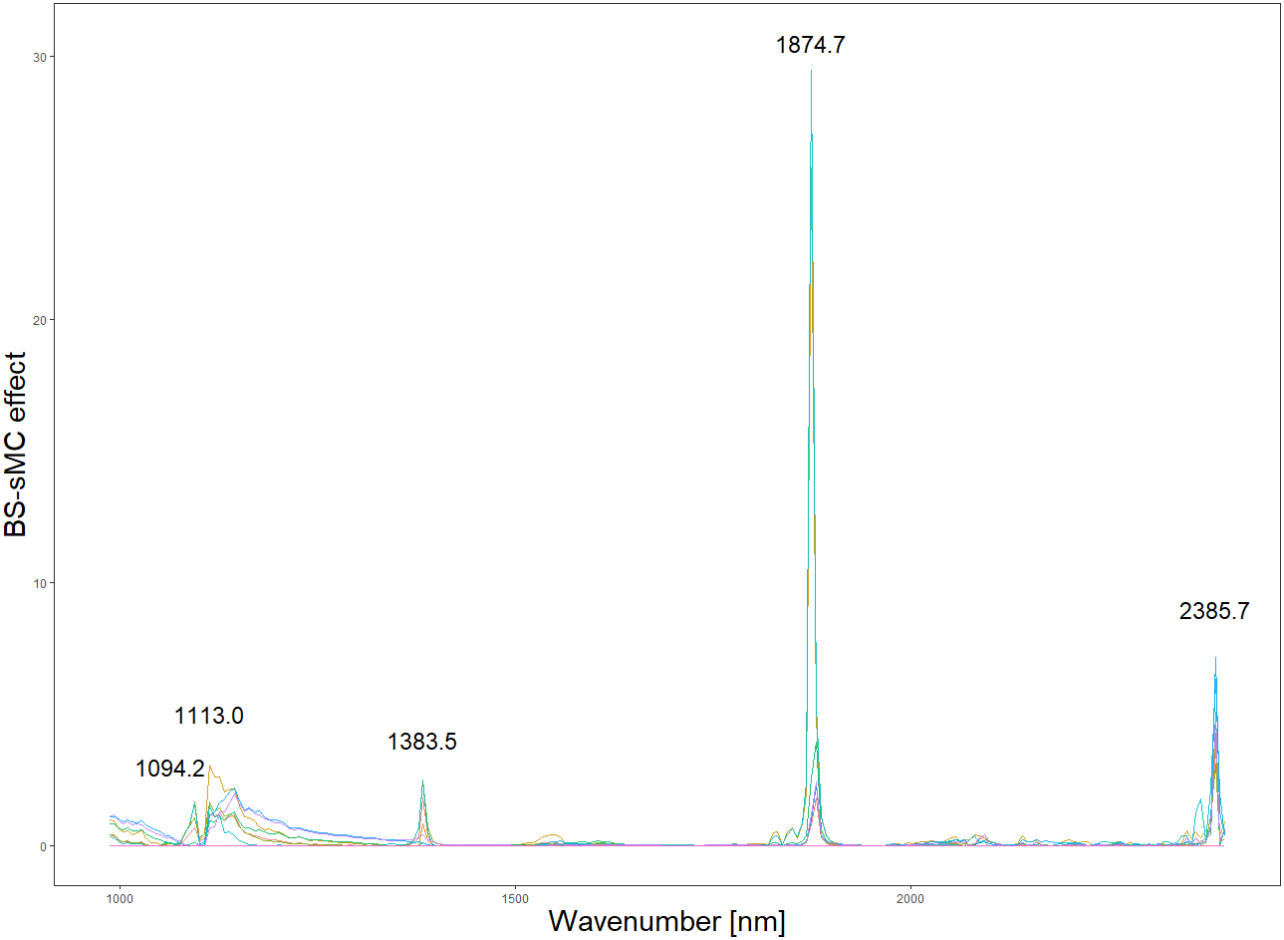
Spectral effects of PLS model with 8 random runs.

Based on the results in **Table 1**, we used the BS preprocessing method and the sMC variable selection algorithm to construct a PLSR model for the prediction of Cy3R content. The constructed Cy3R prediction model was used to estimate the Cy3R content in the validation set, and the estimated values were compared with the actual chemical assay results. As shown in **Figure 3A**, we can observe that the relationship between the estimated values and the actual measured values is closer to a linear regression line, which means that the predicted values in the validation set are closer to the actual values and perform better relative to **Figure 3B**. Therefore, compared to the original full-spectrum model, the Cy3R prediction model utilizes only 9% of the spectral bands to achieve superior prediction results. **Figure 4** illustrates the distribution of residuals for the two models. most of the residual values for the Cy3R prediction model fall in the range of -1 to 1, and only a few residual values are distributed between -2 and 2. Compared to that, most of the residuals of the original full-spectrum model are distributed in the range of -2 to 2. This indicates that the prediction performance of the Cy3R prediction model is more stable and accurate. **Figure 5** shows the eight randomly selected key variables for the Cy3R prediction model when using the sMC variable selection method. Among them, the variables in the bands at 1094.2, 1113, 1383.5, 1874.7, and 2385.7 nm have extremely important effects on the construction of the prediction model. These bands play a key role in the modeling process and help to improve the accuracy and reliability of the predictions.

## 4 Discussion

*M. crassipes*, as an excellent ornamental plant, usually needs to obtain a large amount of trait information during the selection and breeding process. Flower color is an important trait in ornamental plants, which is mainly affected by anthocyanin content (Zhang et al., 2022). Determination of the correlation between flower color phenotype and pigment composition can also provide an important basis for the study of flower color formation mechanism (Fu and Dai, 2016). Although the traditional determination of anthocyanin composition and content has accurate and reliable results, it is time-consuming and destructive to the plant, and it is not possible to monitor the long-term dynamics of a physiological index. Therefore, the aim of this study was to establish a PLSR model using NIR spectroscopy to estimate and predict the Cy3R content of *M. crassipes* tepals, which provides a reference for high-throughput analysis of plant phenotypes. In selective breeding, it is beneficial to obtain the required phenotypic trait information quickly and accelerate the breeding process. One of the most basic and widely used modeling methods for predicting plant physiological content in near-infrared spectroscopy is the partial least squares method. For example, Reuben et al. concluded that the PLSR model could accurately predict the total anthocyanin content of the peel (Buenafe et al., 2022). Olaoluwa et al. accurately predicted avocado ripeness parameters using NIR spectroscopy combined with the PLSR model, and their predictive model for both dry matter and moisture content achieved an R^2^ of 0.92, with RPD values of 2.19 and 2.06, respectively (Olarewaju et al., 2016).

The findings of this study indicate that the utilization of various spectral preprocessing techniques does not uniformly enhance the performance of the models developed. In fact, certain preprocessing methods may result in a diminution of predictive accuracy, aligning with the outcomes reported by Vašát et al. (2017). In this study, we investigated the relationship between NIR spectra and Cy3R content. We compared the performance of prediction models constructed by six different spectral preprocessing methods and four variable selection algorithms in combination with PLSR, and finally confirmed the combination of the BS preprocessing method and the sMC variable selection method as the best prediction model. The R^2^ and RMSE of this model were 0.72 and 1.04%, respectively. These values were lower than the results of Liu et al.’s study (R^2^ = 0.90, RMSE = 0.30%, RPD = 3.19) for the anthocyanin content of *Prunus cerasifera* leaves (Liu et al., 2019). This difference may stem from the different locations where the spectral data were collected. The leaves of *Prunus cerasifera* are relatively large and more easily spreadable, making spectral data collection relatively easy. However, in contrast, *M. crassipes* tepals have a smaller surface area and are irregularly shaped, making them less likely to spread. Therefore, when collecting spectral data from tepals, the fiber-optic probe may not be able to fit completely on their surfaces, which introduces potentially interfering information and reduces the accuracy of the Cy3R content prediction model. In addition, tepals have high moisture content, which may also further reduce the accuracy of the model (Agelet and Hurburgh, 2014; Manzoor et al., 2022).

Models with high R^2^ and low RMSE usually indicate that the difference between the model’s predicted values and the actual measured values is small. However, previous studies have shown that the RPD value is an important indicator for confirming whether a model is reliable or not (Saeys et al., 2005; Davey et al., 2009; Magwaza et al., 2012). It is generally accepted that an RPD value of less than 1.5 implies that the model is unreliable, a model with an RPD value between 1.5 and 2.0 is suitable for rough estimation only, a model with an RPD value between 2.0 and 2.5 is suitable for quantitative prediction, a model with an RPD value between 2.5 and 3.0 is considered good, and a model with an RPD value of more than 3.0 is highly satisfactory (Malley et al., 2000; Saeys et al., 2005; Zimmermann et al., 2007; Magwaza et al., 2012; Olarewaju et al., 2016). In this study, the predictive model built by the BS-sMC combination had an RPD value of 2.06 even though the difference in the R^2^ and RMSE values of the models built by the combination of other different preprocessing and variable selection methods was not considered significant. This means that the model is suitable for quantitative prediction and can be reliably used for prediction of Cy3R content in tepals. This finding proves its potential value in practical applications.

The collection of spectral data is always unavoidably contaminated by environmental noise, so it is important to select effective spectral information (Guo et al., 2020). Appropriate preprocessing of spectral data and variable selection can effectively improve the accuracy of the model and make the modeling task easier (Mishra et al., 2020). The central goal of the BS preprocessing approach is to equalize the effects between different blocks, which may have different scales and number of variables, through block scaling and block variance scaling. This helps to avoid any one block having a dominant influence on the modeling results (Mishra et al., 2021). Analyzing the spectrograms, it is observed that the spectra preprocessed using the BS method exhibit a heightened number of absorption peaks in comparison to spectra treated with alternative preprocessing techniques. This observation might suggest that the BS preprocessing aids in uncovering subtle spectral variances, previously obscured by noise, thereby augmenting the detectability of potential characteristic bands within the spectral data (Vašát et al., 2017). These additional characteristic bands are potentially valuable because they can provide additional quantitative information to the PLSR model. The enrichment of the data has the potential to enhance the stability of the model, as reflected in the significant improvement in the model RPD values. In addition, we found that the sMC algorithm is very effective in variable selection and helps to build a reliable predictive model. This algorithm has been successfully used in other studies to predict different chemical compositions, such as chlorophyll content of *Sassafras tzumu* leaves and malondialdehyde content of slash pine needles (Li et al., 2019; Zhang et al., 2021). sMC algorithm also revealed several important spectral features related to Cy3R in this study, including wavelengths of 1094.2, 1113.0, 1383.5, 1874.7, and 2385.7 nm. As reported by Kokaly et al. phenolics will exhibit spectral features in the range of 1000-1500 nm, with the larger phenolic compounds exhibiting spectral features near 1470 nm, which is caused by the presence of O-H bonds in their molecular structure (Kokaly and Skidmore, 2015). In addition, we observe that the residual values of the model are more tightly distributed within the horizontal bands. This suggests that our predictive model is more suitable for practical applications, as the narrower distribution bands imply better fitting accuracy and higher prediction accuracy. These results further validate the reliability and practicality of our established model.

The model constructed in this study utilizing near-infrared spectroscopy demonstrated promising predictive capabilities; however, there remains scope for further optimization of its performance. Importantly, the dataset acquired reflects merely a single temporal snapshot within a specific year, and the influence of environmental variables (e.g., light and temperature) on the phytochemical composition may introduce additional uncertainty into the predictive model. To enhance the model’s accuracy and reliability, future endeavors will encompass a repeatability assessment and a planned substantial increase in the sample size. These steps will facilitate more comprehensive inversion studies and the subsequent validation of the model’s predictions against laboratory analytical results.

## 5 Conclusions

In this study, a model for predicting the content of cyanidin 3-rutinoside in *M. crassipes* tepals was successfully constructed using NIR spectroscopy and PLSR. This model provides a non-destructive method for the rapid determination of cyanidin 3-rutinoside content in *M. crassipes* tepals. It is worth mentioning that the reliability of the model can be enhanced by using spectral preprocessing and variable selection methods. We clearly demonstrated that the PLSR model based on the combination of the BS preprocessing method and the sMC variable selection method exhibited the best performance. This study not only furnishes essential data for elucidating the biochemical mechanisms underlying flower color formation but also pioneers new pathways for the high-throughput quantitative analysis of flower color phenotypic traits. Moreover, the development of an efficacious predictive model for chemical composition markedly contributes an invaluable reference for the detection and analysis of cyanidin-3-rutinoside content across a broad spectrum of plant research domains, particularly in other plant species.

## Data availability statement

The raw data supporting the conclusions of this article will be made available by the authors, without undue reservation.

## Author contributions

YX: Data curation, Formal analysis, Investigation, Writing – original draft. XZ: Investigation, Writing – original draft. JL: Resources, Supervision, Writing – review & editing. HL: Resources, Writing – review & editing. JJ: Supervision, Writing – review & editing. YL: Conceptualization, Data curation, Writing – review & editing. SD: Conceptualization, Funding acquisition, Project administration, Resources, Writing – review & editing.
